# *MbHY5-MbYSL7* mediates chlorophyll synthesis and iron transport under iron deficiency in *Malus baccata*

**DOI:** 10.3389/fpls.2022.1035233

**Published:** 2022-10-19

**Authors:** Yaqiang Sun, Jiawei Luo, Peien Feng, Fan Yang, Yunxiao Liu, Jiakai Liang, Hanyu Wang, Yangjun Zou, Fengwang Ma, Tao Zhao

**Affiliations:** State Key Laboratory of Crop Stress Biology for Arid Areas/Shaanxi Key Laboratory of Apple, College of Horticulture, Northwest A&F University, Yangling, China

**Keywords:** *Malus baccata*, iron deficiency, chlorophyll synthesis, Fe transporter, regulatory network, *MbHY5*

## Abstract

Iron (Fe) plays an important role in cellular respiration and catalytic reactions of metalloproteins in plants and animals. Plants maintain iron homeostasis through absorption, translocation, storage, and compartmentalization of iron *via* a cooperative regulative network. Here, we showed different physiological characteristics in the leaves and roots of *Malus baccata* under Fe sufficiency and Fe deficiency conditions and propose that *MbHY5* (elongated hypocotyl 5), an important transcription factor for its function in photomorphogenesis, participated in Fe deficiency response in both the leaves and roots of *M. baccata*. The gene co-expression network showed that *MbHY5* was involved in the regulation of chlorophyll synthesis and Fe transport pathway under Fe-limiting conditions. Specifically, we found that Fe deficiency induced the expression of *MbYSL7* in root, which was positively regulated by *MbHY5.* Overexpressing or silencing *MbYSL7* influenced the expression of *MbHY5* in *M. baccata.*

## Introduction

Although iron content is very abundant in the earth, its main existing form is ferric iron (Fe^3+^), which is insoluble and difficult for plants to uptake (Jeong and Guerinot, 2009). Iron (Fe) is one of the most essential micronutrients in plants and plays an important role in whole-life processes, including chlorophyll synthesis, electron transfer, and respiration (Kobayashi and Nishizawa, 2012). Also, iron can affect physiological processes such as nitrogen metabolism, carbohydrate, and organic acid metabolism in plants (Curie and Briat, 2003; Hell and Stephan, 2003; Kobayashi and Nishizawa, 2012).

Fe deficiency can cause a series of problems in fruit production (Tagliavini et al., 1995; Alvarez-Fernandez et al., 2003; Hao et al., 2022). Therefore, revealing the sophisticated mechanism of Fe^2+^ uptake, transport, and homeostasis in fruit plants is important for fruit yield and quality. Fe deficiency affects a variety of physiological and biochemical reactions in the leaves and roots of fruit plants. One of the most prominent symptoms in plant is interveinal chlorosis, or veins yellowing, which leads to a reduced photosynthetic performance of fruit trees (Curie and Briat, 2003; Hao et al., 2022). About 80% of the total iron was stored in chloroplasts; although iron is not a component of chlorophyll, it is an indispensable catalyst for chlorophyll synthesis (Yang et al., 2022). Previous studies have shown that the number of thylakoid membranes decreased in the lamellar structure of the chloroplast under iron deficiency (TerBush et al., 2013). Roots under iron deficiency can form root tip swellings or increase lateral roots and/or root hairs (Morrissey and Guerinot, 2009).

Iron content in plants mainly depends on the uptake and transport of exogenous iron by roots. In plants, there are two distinct strategies for root iron uptaking (Ivanov et al., 2012). Plant species belonging to the dicot and non-graminaceous monocot lineages use Strategy I, which consists of three steps: first, proton efflux from plant cells was mediated by the P-type ATPase to decrease the pH of the rhizosphere soil, which leads to soil acidification and an increase of iron solubility. Meanwhile, Fe(III) is also chelated and mobilized by coumarin-family phenolics exported by an ABC transporter PDR9 from the cortex to the rhizosphere (Tsai and Schmidt, 2017). Next, Fe(III) is reduced to Fe(II) by ferric reduction oxidase 2 (FRO2) localized on the plasma membrane. Third, the divalent iron Fe(II) was taken up into epidermal cells by metal transporter IRT1 (Eide et al., 1996; Santi and Schmidt, 2009). Subsequently, nicotianamine synthase (NAS), yellow stripe-like (YSL), and other transporters helped Fe(II) transport to vacuoles, chloroplasts, and other organs and organelles for further utilization (Walker and Connolly, 2008). Strategy II plants (grasses) synthesize and secrete phytosiderophores (PS) which form chelates with Fe(III) in roots, and this complex was then transported into cells by YSL transporters (Curie et al., 2009). In either way, YSLs play key roles in iron transportation and acquisition. Multiple copies of YSL genes were found in the genomes of angiosperm and gymnosperm species (Chowdhury et al., 2022). *AtYSL1*, *AtYSL3*, *AtYSL4*, and *AtYSL6* have been demonstrated to be involved in the transportation of Fe and Zn from leaves to seeds through the phloem (Murata et al., 2006; Ishimaru et al., 2010; Kumar et al., 2019). The expression of *AtYSL2* was downregulated in response to iron deficiency (Zang et al., 2020). In addition, YSLs have been proposed as transporters of iron from xylem to phloem and then to young tissues (Le Jean et al., 2005; Morrissey and Guerinot, 2009). YSL2 and YSL7 have been found to be associated with the movement of Fe/Zn-NA complexes to maintain Fe homeostasis in *Arabidopsis* (Khan et al., 2018).

HY5 (elongated hypocotyl 5) is a member of the basic leucine zipper (bZIP) transcription factors, which is known for its key roles in light reception and transmission (Gangappa and Botto, 2016; Li et al., 2020). Moreover, HY5 has been shown to be a positive regulator in nitrate absorption, phosphate response, and copper signaling pathways (Zhang et al., 2014; Huang et al., 2015; Chen et al., 2016; Gao et al., 2021). *Arabidopsis* HY5 mutants contain less chlorophyll content (Oyama et al., 1997; Holm et al., 2002; Xiao et al., 2022). A recent study has shown that HY5 can bind the promoter of the FER gene in roots, which is required for the induction of iron mobilization genes, thus providing us a new perspective in understanding the regulatory mechanism of iron uptake in plants (Guo et al., 2021). However, few studies have reported the correlation of HY5 and chlorophyll synthesis genes under Fe-deficient conditions. Moreover, no report has yet been published on the regulative role of HY5 to YSL iron transporters in response to iron stress in *Malus*.

*Malus baccata* has been widely used as a cold-resistant apple rootstock, especially in Northeast China. However, *M. baccata* is sensitive to iron deficiency. In this study, we compared the physiological characteristics and the transcriptive features of *M. baccata* under Fe-sufficient/deficiency conditions in the leaves and the roots and explored the regulative role of MbHY5 to chlorophyll metabolic genes and iron transporters (*MbYSL*). Our results provide insight into the molecular mechanism of iron deficiency response in *M. baccata*.

## Materials and methods

### Plant material and growth conditions

*M. baccata in vitro* shoots were cultured on MS medium (0.5 mg/l 6-BA and 0.5 mg/l IBA) for 30 days (Hao et al., 2022). Next, seedlings (with a height ~5 cm) were transported to the rooting medium (0.5 mg/l IBA) and cultured for 30 days. Rooted seedlings were transplanted into an improved-Hoagland nutrient solution and cultured for 3 weeks. Seedlings were cultivated at 25 ± 2°C day/21 ± 1°C night with a 16-h day/8-h night photoperiod.

### Measurement of chlorophyll contents and rhizosphere pH

Seedling leaves grown on Fe-sufficient (+Fe, 40 μM) and Fe-deficient (-Fe, 0 μM) for 0, 24, 72, and 144 h were sampled, respectively. Leaves were cut into pieces after cleaning and removal of the veins. Next, 0.2 g tissues was mixed with quartz sand, calcium carbonate, and 95% ethanol. The absorbance of the filtrate was measured using a spectrophotometer (Shimadzu, Kyoto, Japan) at 663 and 645 nm. The rhizosphere pH was measured using a pH meter.

### FCR activity

FCR activity was determined by the Ferrozine assay. The roots were first cultivated under +Fe and -Fe conditions for 0, 72, and 144 h and were then submerged into a chromogenic medium (0.5 mM ferrozine, 0.5 mM FeNa-EDTA, 0.5 mM CaSO_4_, and 0.7% (w/v) agar (Schmidt et al., 2000)) and incubated in the dark for 1 h. All measurements were performed at room temperature with a Shimadzu spectrophotometer (Kyoto, Japan).

### Perls staining

Fresh root, stem, and leaf tissues were collected and placed in a small box (2 cm*2 cm*2 cm), which contains an appropriate amount of OCT, with tissues submerged by an embedding agent. Next, the bottom of the box was exposed to liquid nitrogen for quick freezing. Finally, the embedded blocks were placed on a freezing microtome for slicing, with continuous slicing of 10~20 μm. Perls staining was conducted using a Prussian Blue Iron Stain Kit (Solarbio, 60533ES20). Micro-tissues were transferred into Perls solution and stained for 0.5~1 h, then they were washed with deionized water and incubated in the methanol solution (Sun et al., 2020). Imaging was performed with a volume microscope (BA210, Motic) (Jia et al., 2018).

### Fe content

The roots and leaves of the *M. baccata* seedlings treated under +Fe and -Fe conditions (see above) at different times were sampled 1 g for each sample. The samples were first dried at 105°C for 30 min then were placed at 80°C for 72 h till the samples were completely dry. Inductively coupled plasma–optical emission spectrometry was used to determine the active iron contents (Zheng et al., 2021).

### Quantitative real-time PCR and public RNA-seq data analysis

Total RNA was extracted from the roots of *M. baccata* seedlings and was purified using the RNAprep Pure Plant Kit (TIANGEN, Beijing, China) according to the manufacturer’s instructions. cDNA was prepared from total RNA using the HiScript II 1st Strand cDNA Synthesis Kit (+gDNA wiper) (Vazyme, Nanjing, Chain). The LightCycler^®^ 480 II system (Roche) was used for the qPCR assay, and the primers are listed in **Supplementary Table 5**. The relative expression of each gene was calculated based on the 2^-△△Ct^ method.

A total of 30 groups of RNA-seq data from a project (PRJNA598053) was used to analyze the expression pattern of chlorophyll synthesis and iron transporter genes under Fe sufficiency and Fe deficiency conditions (0, 24, and 72 h) (Sun et al., 2020) (https://www.ncbi.nlm.nih.gov/bioproject/PRJNA598053/ ). Data for the project were downloaded from the NCBI database, including roots and leaves. The expression abundance of the leaves and roots genes was calculated using the FPKM value, and the relative expression level is shown as log2 (fold change) values.

### Plasmid construction and GUS histochemical staining

The full length of the *MbHY5*-coding sequence was inserted into the PRI101 (AN) vector. The promoters (upstream ~2 kb) of *MbYSL7* or *MbYSL2* were cloned respectively into the pCAMBIA1391 vector with the GUS reporter (Li et al., 2021b). Histochemical GUS staining of *Nicotiana benthamiana* leaves was conducted as previously described (Liu et al., 2002; Sun et al., 2020). The samples were incubated for 24 h at 37°C. Chlorophyll was removed by washing the samples with 70% (v/v) ethanol for 2 days. Imaging was performed with a volume microscope (MZ10F, Leica).

### Transient expression

The full length of the *MbYSL7*-coding sequence was amplified without the stop codon using the specific primer pairs (**Supplementary Table 5**) and was inserted into the PRI101 (AN) vector with the *35S* promoter. In order to repress the expression of *MbYSL7*, the pTRV*-MbYSL7* vector was constructed as previously described (Sun et al., 2020; Hao et al., 2022). The *MbYSL7-*overexpression and VIGS vectors were transformed into *Agrobacterium tumefaciens* cells (GV3101). Infected apple seedlings were placed in a dark place for 2 days and then were transferred to normal light conditions for 1 day. Seedlings grown on Fe-sufficient and Fe-deficient conditions for 0, 24, 72, and 144 h were sampled and then stored at -80°C for RNA extraction.

### Yeast one-hybrid assay

The full-length *MbHY5* CDS sequence was inserted into pB42AD (AD vector), while the MbYSL7 or MbYSL2 protein-binding sites (CACGTG) were inserted into pLacZi (BD vector). The fusion vectors were transformed into the yeast EYG48 strain (Li et al., 2021b; Wu et al., 2021).

### Phylogenetic tree

Homologous YSL gene sequences of *M. domestica*, *M. baccata*, and *Arabidopsis thaliana* were aligned using ClustalX version 2.0 (Jeanmougin et al., 1998). The phylogenetic tree was constructed in MEGA (version 11) (Tamura et al., 2021) with the Neighbor-Joining method (bootstrap replicates = 100).

### Co-expression gene network analysis

In order to identify key genes involved in Fe deficiency in *M. baccata*, chlorophyll synthesis-related genes and iron homeostasis-related genes were selected, and their expression patterns under Fe deficiency were investigated based on the transcriptome data. Subsequently, their co-expressed genes were predicted using the AppleMDO database (network analysis) (http://bioinformatics.cau.edu.cn/AppleMDO/) (Da et al., 2019). Finally, these genes (503 genes in the leave samples and 693 genes in root samples) were selected to construct the co-expression network using Cytoscape 3.8.0 (Shannon et al., 2003; Zhao et al., 2017).

### Statistical analysis and diagram drawing

Statistical analyses were executed using GraphPad Prism. The correlation of MbHY5 and chlorophyll synthesis- and roots iron homeostasis-related genes was calculated using the Pearson correlation (Lv et al., 2021). All statistical analyses were performed by one-way ANOVA test, with *p* ≤ 0.05 considered as significantly different among different samples. Diagrams illustrating the mechanism of chlorophyll synthesis and Fe acquisition were created using BioRender (https://biorender.com/) (Therby-Vale et al., 2022).

## Results

### *M. baccata* leaves and roots are sensitive to Fe deficiency

**Figure 1:**
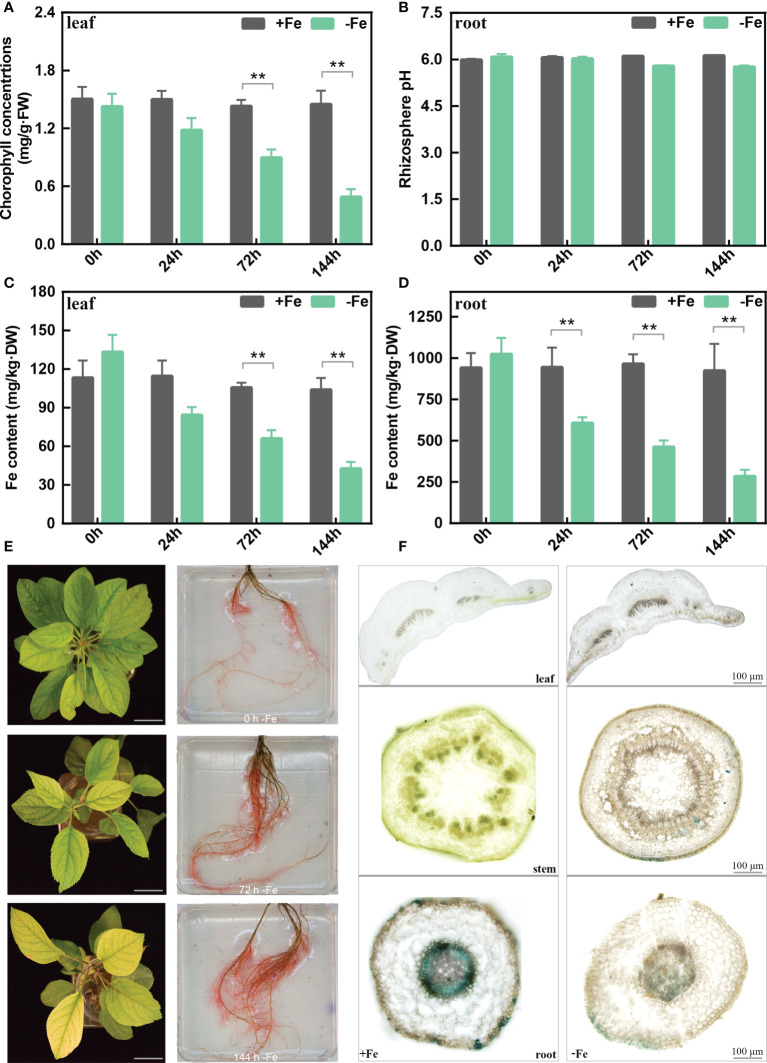
The physiological changes in the leaves and roots of *M. baccata* seedlings under Fe-deficient and Fe-sufficient conditions. **(A)** Chlorophyll concentration in leaves. **(B)** Rhizosphere pH in roots. **(C)** Fe content in leaves. **(D)** Fe content in roots. **(E)** Chlorosis extent in leaves and corresponding FCR activities in roots under 0, 72, and 144 h (scale bar: 0.5 cm). **(F)** Perls staining of different tissues, including leaf, stem, and root (scale bar: 100 μm). Asterisks indicate statistically significant differences (**p < 0.01). Error bars denote ± SD (biological replicates = 3).

The chlorophyll content of *M. baccata* leaves showed a continual decrease from 0 to 144 h (**Figure 1A**) under -Fe treatments. After 144 h, the rhizosphere pH of -Fe treatment was lower than that of +Fe treatment, but with no statistically significant differences (**Figure 1B**). The results indicated that iron deficiency caused lower chlorophyll content in the leaves and a decrease in rhizosphere pH. Meanwhile, as for the content of active Fe in the leaves, it decreased from 104 to 42 mg/kg·DW after 144-h Fe deficiency stress. Similarly, its content in the roots also decreased from 923 to 284 mg/kg·DW (**Figures 1C, D**).

We further measured the FCR activity of the roots to better understand the iron acquisition processes. Fe-deficient roots showed higher FCR activity in contrast with Fe-sufficient roots at different treatment times (**Figure 1E**). Moreover, Perls staining results showed that tissues (leave, stem, and root) from Fe-sufficient conditions showed stronger Fe^3+^ staining than Fe-deficient ones (**Figure 1F**). Interestingly, it also showed that Fe deficiency induces a sharp decrease of Fe^3+^ in xylem and phloem (**Figure 1F**). In conclusion, these results revealed that iron deficiency induced morphological and biochemical changes in *M. baccata*, including decreases in chlorophyll content, rhizosphere pH, and active iron content in the leaves and roots.

### Iron deficiency induced the expression of chlorophyll synthesis genes in leaves

**Figure 2:**
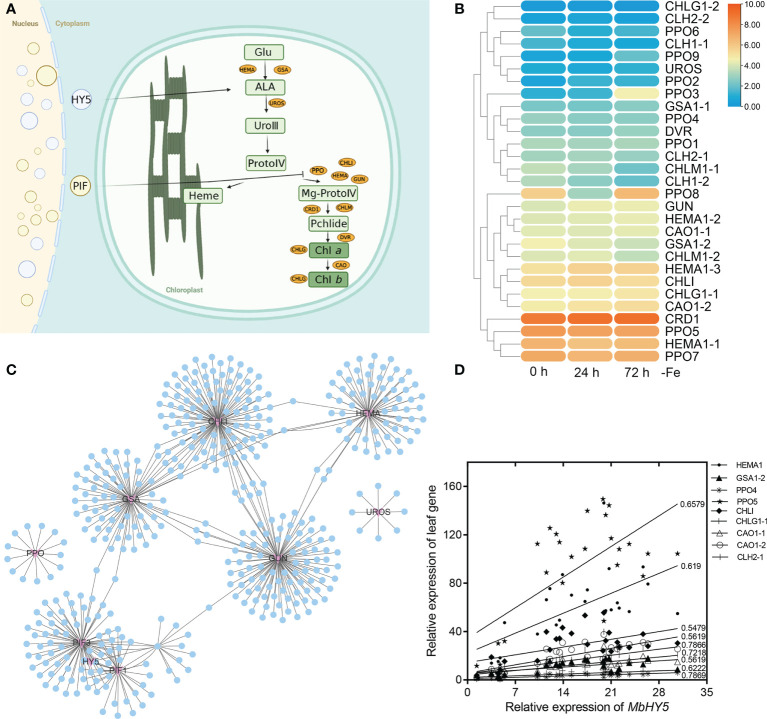
*MbHY5* was associated with the chlorophyll synthesis genes in the leaves under Fe deficiency. **(A)** The assumption model of the *MbHY5* gene participating the regulation of chlorophyll synthesis. Ovals represent chlorophyll biosynthesis-related genes. HEMA: glutamyl-tRNA reductase, GSA: glutamate-1-semialdehyde 2,1-aminotransferase, CHLH, Mg-chelatase, CHLM, Mg-protoporphyrin IX methyltransferase, CRD, Mg-protoporphyrin IX monomethylester cyclase; DVR, divinyl chlorophyllide a 8-vinyl-reductase; CAO, Chla oxygenase; CHLG, chlorophyll synthase; PPO, protochlorophyllide oxidoreductase. The model was drawn by BioRender (https://biorender.com/ ). **(B)** The heatmap showing the expression of chlorophyll biosynthesis-related genes in Fe-deficient conditions. **(C)** The co-expression network of HY5/PIF and chlorophyll biosynthesis-related genes. **(D)** Pearson correlation coefficients between *MbHY5* and chlorophyll biosynthesis-related genes.

We hypothesized that the well-known light-responsive gene *HY5* or *PIF* genes may have participated in the regulation of the chlorophyll synthesis process (**Figure 2A**). Indeed, we detected a series of chlorophyll metabolic genes from RNA-seq analysis under Fe deficiency, including Glu-tRNA reductase (*HEMA*), Glu 1-semialdehyde (*GSA*), uroporphyrinogen III synthase (*UROS*), chlorophyll synthase (*CHLG*), GUN, Chla oxygenase (*CAO*), protochlorophyllide oxidoreductase (*PPO*), and divinyl reductase (*DVR*). The results showed that *HEMA1-1*, *HEMA1-2*, *CHLG1-1*, *CHLI*, *PPO5*, *CAO1-2*, and *CRD1* were highly expressed in all treatment times (**Figure 2B**). In contrast, the gene expressions of *DVR*, *CHLG1-2*, *CLH1-1*, *UROS*, and *CLH* were significantly lower in leaves (**Figure 2B**). Specially, the expression levels of PPO3, PPO9, CHLM1-1, CLH1, PPO8, GUN, GSA1-2, CHLM1-2, HEMA1-3, CAO1-2, CHLG1-1, and HEMA1-1 were significantly changed under Fe deficiency. We constructed a gene co-expression network to investigate the correlation of HY5 or PIF genes and the chlorophyll biosynthesis-related genes. The results showed that *HY5*, *PIF1*, *PIF3*, *HMEA*, *GSA*, and *GUN* form a complicated co-expression network in regulating chlorophyll biosynthesis (**Figure 2C**; **Supplementary Table 1**). Moreover, the expression levels of *HY5* were positively correlated with those of most chlorophyll biosynthesis-related genes, such as *HMEA*, *GSA1-2*, *CAO*, *CHLI*, *PPO*, and *GUN*. The Pearson correlation coefficients between *HY5* and these genes ranged from 0.54 to 0.78. In contrast, *UROS*, *CHLG1-2*, *DVR*, and *CLH1-1* were only slightly correlated or did not correlate with *HY5* (**Figure 2D**).

### Analysis of the expression profiles of Strategy I-related genes under iron deficiency

**Figure 3:**
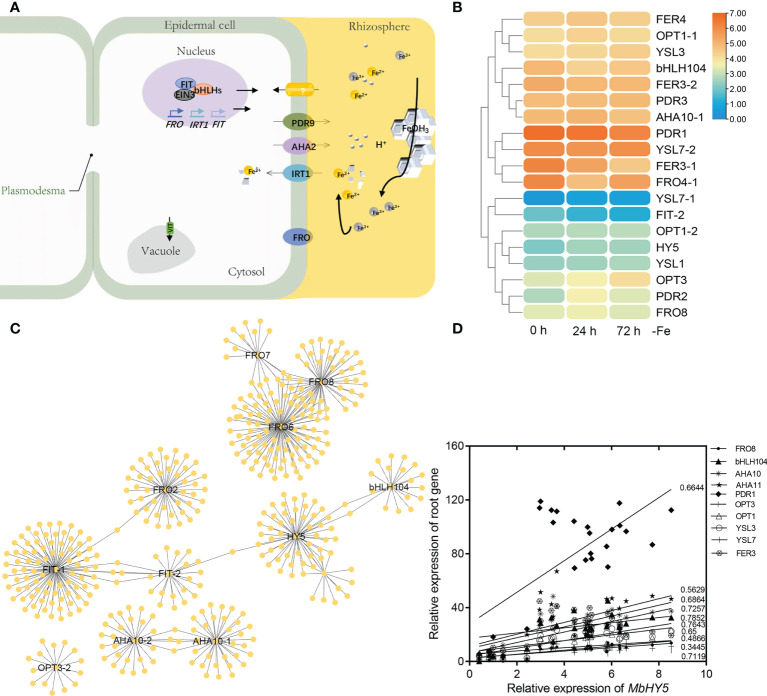
MbHY5 is related with Fe acquisition and transportation-related genes in roots in Fe-deficient conditions. **(A)** A model showing the acquisition and transportation of Fe in the roots of dicot plants under Fe deficiency. Ovals represent Fe homeostasis-related genes. **(B)** The heatmap showing the expression levels of the Fe homeostasis-related genes. **(C)** The co-expression network of HY5 and iron responsive genes in roots. **(D)** Pearson correlation coefficients between *MbHY5* and Fe homeostasis-related genes.

Under iron deficiency conditions, *Malus baccata*, similar to other dicots, use Strategy I to acquire Fe in roots. We summarized the key genes reported in transferring and regulating Fe^2+^ transportation from the rhizosphere into root cells, including *AHA2*, *FRO2*, *PDR9*, *IRT1*, *bHLH100/101*, *OPT3*, and *FIT* (Ito and Gray, 2006; Satbhai et al., 2017; Khan et al., 2018; Lv et al., 2021; Pei et al., 2022) (**Figure 3A**). Under Fe deficiency, most of these genes were highly induced in roots, especially for *PDR1*, *HY5*, *YSL7*, *FDR2*, and *FER* genes (**Figure 3B**).

In order to analyze the regulatory network of iron homeostasis genes in roots, a total of 693 iron homeostasis-related genes in roots were selected to construct the co-expression network, and the results showed that *MbHY5*–*bHLH04*–*FIT*–*FRO2* constructed the biggest module, indicating that MbHY5 plays an essential role in regulation iron homeostasis in roots (**Figure 3C**; **Supplementary Table 2**). Pearson correlation analysis further showed that iron homeostasis-related genes differentially expressed in root under Fe deficiency were significantly positively related with MbHY5, including OPT3, PDR1, bHLH104, YSL, and AHA10 (**Figure 3D**). The correlation coefficients ranged from 0.45 to 0.78 (**Figure 3D**).

### MbHY5 directly promotes the expression of *MbYSL7* in response to Fe deficiency

**Figure 4:**
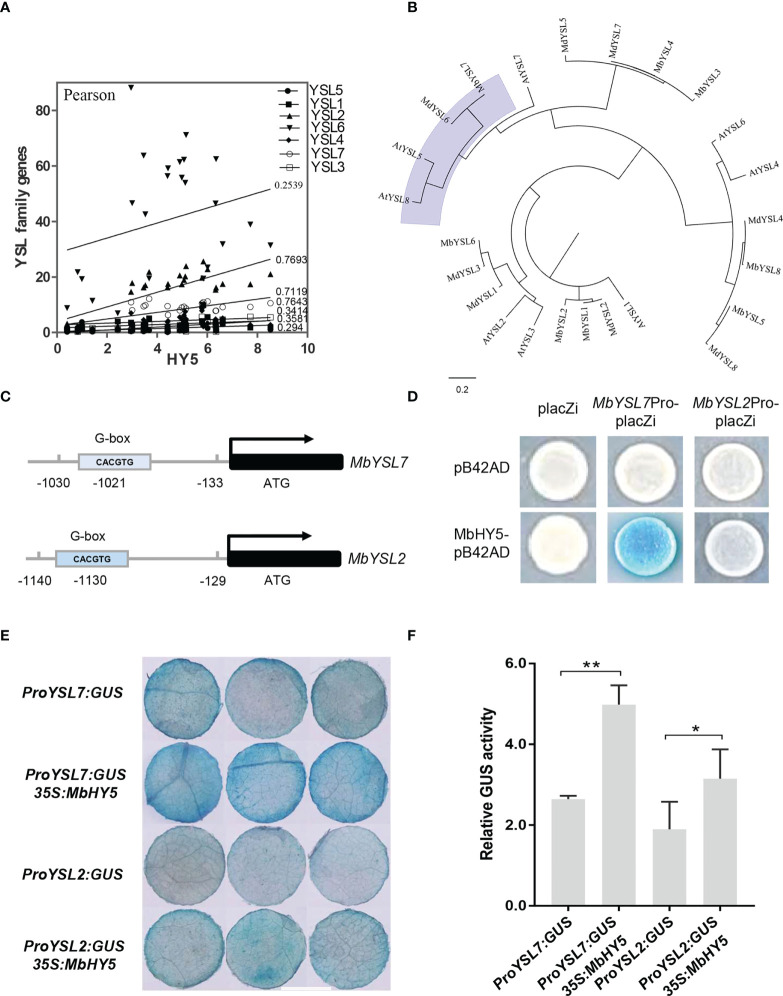
*MbHY5* regulates *MbYSL7* under Fe deficiency. **(A)** Pearson correlation coefficients of *MbHY5* and YSL family genes. **(B)** The phylogenetic tree of the YSL proteins in three species, including *Arabidopsis*, *M. baccata*, and *M. domestica*. **(C)** Putative HY5-binding site (G-box) was found in the promoter region of *MbYSL7* and *MbYSL2*, respectively. **(D)** Y1H assay. Coding sequence of *MbHY5* was inserted into pB42AD while the promoter region of MbYSL7 or MbYSL2 was inserted into pLacZi, respectively. **(E, F)** GUS staining and GUS enzyme activity of transient transformations of tobacco leaves (scale bar = 1 cm); gene constructs used for the transformations were labeled. Error bars indicate SDs (biological replicates = 3), asterisks indicate statistically significant differences (*p < 0.05, **p < 0.01).

We found that the expressions of *YSL2* and *YSL7* were highly related to HY5 (r = 0.7693 and 0.7119, respectively, Pearson correlation) (**Figure 4A**). The phylogenetic tree showed that each of the apple YSL genes clustered with its closely related homologous genes in *Arabidopsis* (**Figure 4B**). Previous studies have shown that HY5 can bind to the promoters of *SlFER* and *AtBTS* and induce the expression of a series of iron-uptaken genes under iron-deficient conditions (Guo et al., 2021; Mankotia et al., 2022).

A G-box (CACGTG) element was found in each of the promoters of *MbYSL2* and *MbYSL7*, which allows HY5 binding (**Figure 4C**). Y1H analysis showed that MbHY5 can directly bind to the promoter of *MbYSL7*, but not that of *MbYSL2* (**Figure 4D**). Transient transformation of tobacco leaves with *proMbYSL7:GUS* showed lower GUS activity than co-transformation with *35S:MbHY5* (**Figures 4E, F**). Similarly, co-transformation of *35S:MbHY5* and *proMbYSL2:GUS* showed slightly higher GUS activity than the transformation of *proMbYSL2:GUS* only (**Figures 4E, F**). In conclusion, these data suggested that *MbHY5* functions as a positive and direct regulator of *MbYSL7*.

### Expression of *MbYSL7* in transient transgenic apple seedlings

**Figure 5:**
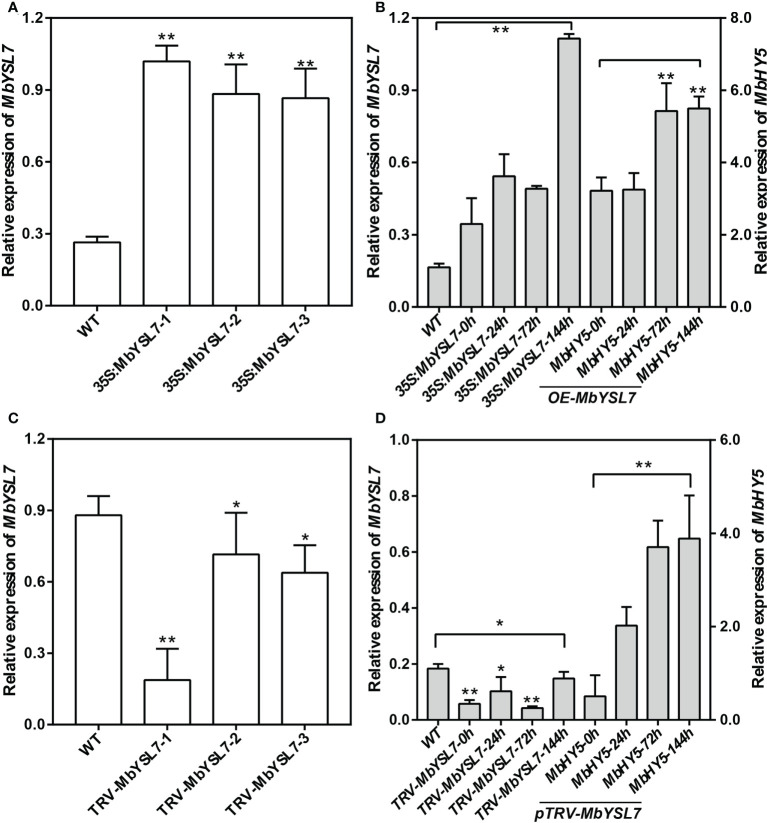
Expression of *MbHY5* in transient transgenic *M. baccata* seedlings overexpressing or silencing *MbYSL7.* **(A)** Relative expression levels of *MbYSL7* in transgenic lines overexpressing *MbYSL7*. **(B)** Relative expression levels of *MbYSL7* and *MbHY5* in the roots of the overexpression lines under Fe deficiency. **(C)** Relative expression levels of *MbYSL7* in transgenic lines silencing *MbYSL7*. **(D)** Relative expression levels of *MbYSL7* and *MbHY5* in the roots of the overexpression lines under Fe deficiency. Error bars indicate SDs (biological replicates = 3), asterisks indicate statistically significant differences (*p < 0.05, **p < 0.01).

To further investigate whether *MbYSL7* was involved in regulating Fe deficiency responses in apple, we made transient transformed lines of apple seedlings with overexpression vector and VIGS vector, respectively. As we can see, compared with the control line, the expression levels of *MbYSL7* were highly induced in the transient transformed apple seedling lines of *35S:MbYSL7*-1, -2, and -3 (**Figure 5A**). Under –Fe treatment, the expressions of *MbYSL7* and *MbHY5* were highly increased in *MbYSL7* overexpression lines, compared with the control lines (**Figure 5B**). The expression of *MbYSL7* was greatly reduced in *pTRV : MbYSL7*-1 (**Figure 5C**). Specifically, the expression of *MbYSL7* slightly increased at the 144-h -Fe treatment, compared with that of the 72-h treatment. In comparison, the expression level of *MbHY5* was lowest at the initial -Fe treatment but greatly induced from 24 h onward (**Figure 5D**). Similar to *MbHY5*, we found that *MbYSL7* was positively related with chlorophyll synthesis-related genes as well, including *PPO5*, *GSA1-2*, and *HEMA* (**Supplementary Table 3**). In addition, we observed that *MbYSL7* positively correlated with most of Fe homeostasis genes in root either, such as *AHA10*, *bHLH104*, and *PDR2*; the correlation coefficients ranged from 0.40 to 0.92 (**Supplementary Table 4**).

## Discussion

In plants, iron deficiency leads to chlorosis caused by a reduced chlorophyll biosynthesis (Li et al., 2021a). Chlorophyll content decreased dramatically in chlorosis leaves under Fe deficiency (**Figure 1**), which is in agreement with the findings in citrus and grapes (Chen et al., 2004; Jin et al., 2017). Iron deficiency increased ferric chelate reduction (FCR) activity and decreased the rhizosphere pH of the apple roots (**Figure 1**). Also, we observed a reduction of active Fe content in the leaves and roots under iron deficiency. Perls staining is a reliable chemical method to stain the iron trivalent in tissues; ferric iron reacts with potassium ferrocyanide and generates blue insoluble compounds (Lv et al., 2021; Hao et al., 2022). Under Fe deficiency, a lower ferric iron content was observed compared to that of the Fe-sufficient treatment (**Figure 1**).

HY5 has been found to be involved in the metabolism of nitrogen (N), phosphorus (P), copper (Cu), sulfur (S), etc. (Zhang et al., 2014; Gangappa and Botto, 2016; Yang et al., 2020; Gao et al., 2021). In *Arabidopsis*, HY5 regulates the expression of key nitrogen signaling genes including *NIA1*, *NIR1*, *NRT1.1*, *NRT2.1*, and *AMT1;2* (Jonassen et al., 2008; Jonassen et al., 2009; Yanagisawa, 2014; Chen et al., 2016; Xiao et al., 2022). In apple, *NIA2* and *NRT1.1* were positively regulated by HY5 in promoting nitrate assimilation (An et al., 2017). Nevertheless, few studies have reported its function in Fe uptake and homeostasis. In *Arabidopsis*, HY5 regulates *BTS* in response to Fe deficiency. Similar results were also found in tomato, in which the *HY5*-*FER* pathway could be involved in Fe metabolism (Guo et al., 2021; Mankotia et al., 2022). In the present study, we firstly found that *MbHY5* was significantly changed in *M. baccata* under Fe deficiency. HY5 plays essential roles in photosynthetic pigment synthesis in light responses (Liu et al., 2017; Liu et al., 2020). It regulates the expression of chlorophyll-related genes in leaves, including *HEMA1*, *GUN4*, *CAO*, *PORC*, and *CHLH* (Toledo-Ortiz et al., 2014; Job and Datta, 2021). In addition, HY5 can regulate the genes involved in maintaining iron homeostasis, such as *FRO2*, *FIT*, *IRTI*, and *PYE* in roots (Mankotia et al., 2022). Further analysis found that *MbHY5* participated in the regulation of chlorophyll synthesis in the leaves and iron acquisition in the roots under iron deficiency (**Figures 2** and **3**). Our results enriched the regulatory mechanism of HY5 in plants in response to Fe deficiency.

**Figure 6:**
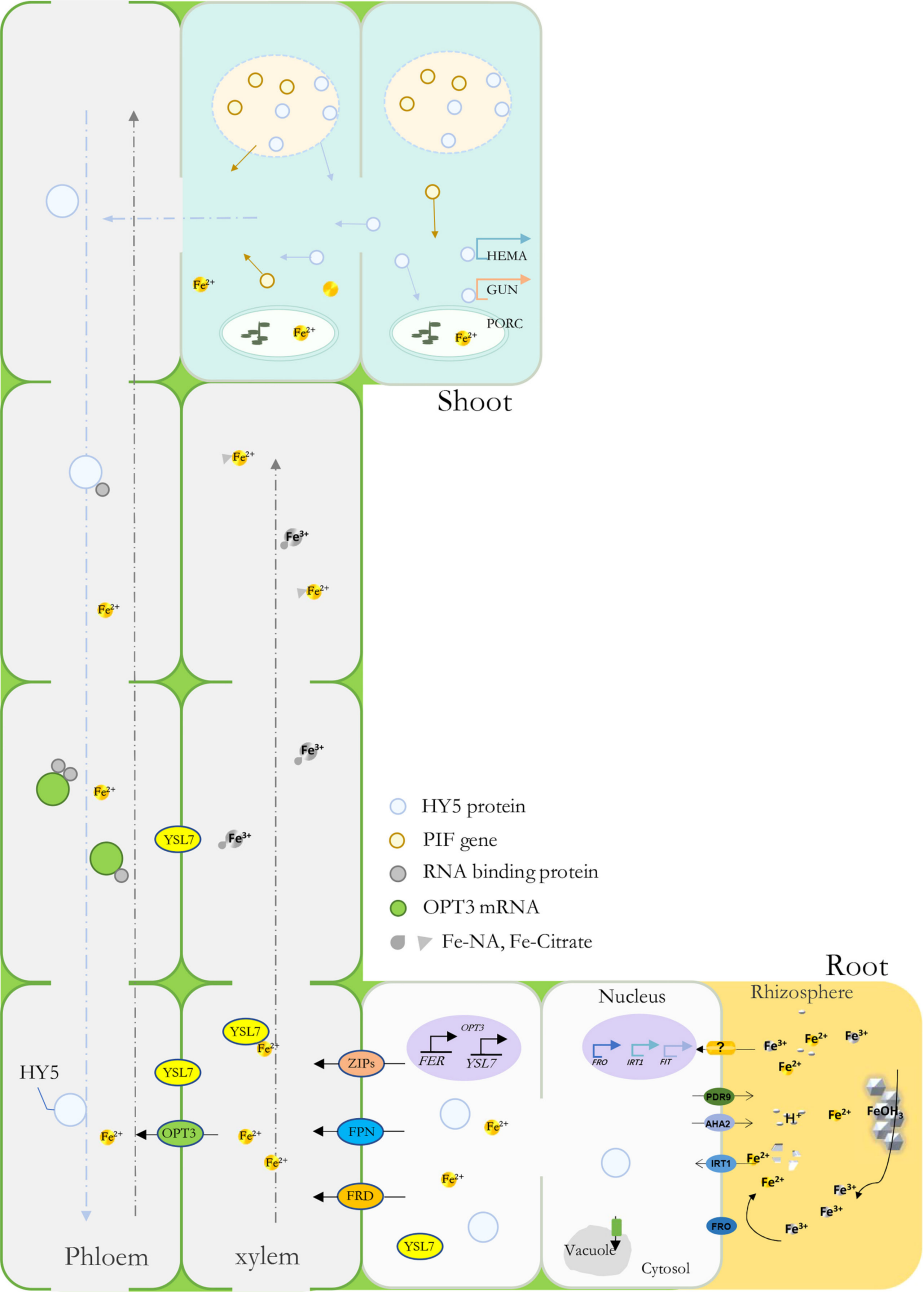
A model depicting MbHY5 as an important regulative transcription factor by regulating chlorophyll synthesis-related genes in the leaves, and Fe acquisition and transportation-related genes in the roots under Fe deficiency.

YSL genes have been found to participate in plant metal uptake, such as Cu and Fe (Ishimaru et al., 2010; Zheng et al., 2012; Dai et al., 2018). In *Arabidopsis*, *AtYSL1-3* and *AtYSL6-8* were responsive under Fe deficiency conditions; among them, some were characterized as long-distance signaling media or Fe(II)-NA transporters (Waters et al., 2006; Castro-Rodriguez et al., 2021). Previously, *MtYSL7*, *AtYSL7*, and *GmYSL7* were identified and characterized as peptide transporters without further functional annotation (Castro-Rodriguez et al., 2021; Gavrin et al., 2021). Our results suggested that *MbYSL7* plays an important role under Fe deficiency. Interestingly as evidenced by our Y1H and the transient co-transformation assays, *MbYSL7* was positively regulated by MbHY5. Overall, we propose that MbHY5-YSL7 was involved in regulating the genes involved in chlorophyll synthesis and iron transportation, in both the leaves and the roots, to alleviate iron deficiency-caused chlorosis and to promote Fe transportation (**Figure 6**).

## Conclusion

Contrasting differences of chlorophyll content and the concentration of active iron were observed under +Fe and -Fe conditions in *M. baccata*. We propose that MbHY5 functions as a vital transcription factor in regulating chlorophyll synthesis and Fe transportation. Lastly, MbHY5 directly regulates the expression of *MbYSL7* in roots under Fe deficiency.

## Data availability statement

The datasets presented in this study can be found in online repositories. The names of the repository/repositories and accession number(s) can be found in the article/**Supplementary Material**.

## Author contributions

YS designed the experiment, analyzed the data, and drafted the manuscript. YS, JWL, and PF prepared the materials and performed the bioinformatics analysis. YL, JKL, and FY helped with the qRT-PCR analysis. YZ, FM, and TZ edited the manuscript. All authors contributed to the article and approved the submitted version.

## Funding

This work was financially supported by the National Natural Science Foundation of China (32102311 and 32102338), the China Postdoctoral Science Foundation (2021M690129), the Chinese Universities Scientific Fund (2452020265 and 2452021133), and the Xinjiang Production and Construction Corps Key Laboratory of Protection and Utilization of Biological Resources in Tarim Basin (BRZD2105).

## Conflict of interest

The authors declare that the research was conducted in the absence of any commercial or financial relationships that could be construed as a potential conflict of interest.

## Publisher’s note

All claims expressed in this article are solely those of the authors and do not necessarily represent those of their affiliated organizations, or those of the publisher, the editors and the reviewers. Any product that may be evaluated in this article, or claim that may be made by its manufacturer, is not guaranteed or endorsed by the publisher.
